# Real-world utilization patterns and outcomes of colesevelam hcl in the ge electronic medical record

**DOI:** 10.1186/1472-6823-13-24

**Published:** 2013-07-17

**Authors:** Richard A Hansen, Joel F Farley, Matthew L Maciejewski, Xin Ye, Chunlin Qian, Ben Powers

**Affiliations:** 1 Department of Pharmacy Care Systems, Harrison School of Pharmacy, Auburn University, Auburn, AL, USA; 2 Division of Pharmaceutical Outcomes and Policy, University of North Carolina Eshelman School of Pharmacy, Chapel Hill, NC, USA; 3 Center for Health Services Research in Primary Care, Durham VA Medical Center, Durham, NC, USA; 4 Division of General Internal Medicine, Duke University, Durham, NC, USA; 5 Daiichi-Sankyo, Inc, Parsippany, NJ, USA; 6 St. Luke’s Health System, Boise, ID, USA

## Abstract

**Background:**

In randomized controlled trials (RCTs), colesevelam HCI, added to other anti-diabetic therapy, reduced hemoglobin A1C by approximately 0.3% to 0.4% over 16- to 26-weeks compared with an increase of approximately 0.1% to 0.2% for placebo, for a placebo-adjusted treatment effect of approximately 0.5%. Evidence on real-world effectiveness is unknown. This retrospective cohort study examined A1C changes following colesevelam HCL initiation in patients with diabetes, regardless of concomitant anti-diabetic medication use.

**Methods:**

2000–2011 GE Centricity electronic medical records data were used to identify patients with type 2 diabetes mellitus (T2DM) aged 18 or older initiating colesevelam HCL. The sample was further restricted to uncontrolled patients with database activity ≥ 395 days before and after colesevelam HCL initiation, A1C > 7% during 90 days prior to starting colesevelam HCL, without prior use of bile acid sequestrants, and with at least one A1C result between 42 to 210 days after initiation. Three overlapping time intervals were created for A1C measurement, including 16-weeks, 26-weeks, and 52-weeks following therapy initiation. The last observed A1C lab measurement during each interval was used to define change from baseline. Mean change in A1C was examined using paired t-tests. Sensitivity analyses considered only patients who remained on colesevelam HCL through each respective measurement period, as well as the effect of concomitant diabetes medications.

**Results:**

Of 1,709,393 patients in the GE database with T2DM, 1,747 met inclusion criteria. The cohort was 58% female, 38% age ≥ 65, and the majority was white. For the 16-week endpoint (N = 1,385), A1C dropped from a mean of 8.22% to 7.75% (mean change −0.47%; P < 0.0001). For the 26- and 52-week endpoints (N = 1,747), A1C dropped from a mean of 8.25% to 7.81% (mean change −0.44%; P < 0.0001) and 8.25% to 7.79% (mean change −0.46%; P < 0.0001), respectively. Sensitivity analyses showed that A1C reductions were of similar direction and magnitude for patients who remained on treatment, and for the subgroups of patients stratified by receipt of concomitant T2DM treatments.

**Conclusions:**

The 0.44% to 0.47% A1C reduction observed in this study was similar to the reduction observed in RCTs, supporting the real-world effectiveness of colesevelam HCL in reducing A1C.

## Background

Diabetes is a costly and increasingly prevalent condition with the number of affected people expected to reach 366 million worldwide by 2030, representing more than a 2-fold increase from the 171 million people with diagnosed diabetes in 2000 [1]. Effective prevention and treatment of diabetes is becoming more critical than ever because the diabetes risk factor of obesity is increasing in prevalence and diabetes is now the seventh leading cause of death in the U.S. Diabetes is also the leading cause of kidney failure, non-traumatic lower-limb amputation, and new cases of blindness among U.S. adults [2].

Appropriate management of glycemic control in type 2 diabetes mellitus (T2DM) can reduce risks of retinopathy, neuropathy, nephropathy, and mortality [3,4]. Given that cardiovascular disease is strongly implicated in diabetes-related mortality [5], managing other cardiovascular risk factors is especially important for these patients [6-8]. In addition to lifestyle modifications to address weight, smoking, dietary intake, and physical activity, controlling diabetes with medications is an important aspect of preventing diabetes morbidity and mortality [7,9].

In late 2000, colesevelam HCL (Welchol) was approved by the U.S. Food and Drug Administration (FDA) as an adjuvant therapy to manage diabetes. Colesevelam HCL is a second-generation bile acid sequestrant that, in addition to diet and exercise, can reduce low-density lipoprotein cholesterol (LDL) in patients with hyperlipidemia, and reduce glycated hemoglobin (A1C) in patients with T2DM. Three randomized trials demonstrated A1C improvements ranging from 0.50% to 0.54% when patients on oral agents or insulin were augmented with colesevelam HCL compared with placebo [10-12]. These randomized controlled trials have assessed the addition of colesevelam HCL to metformin over 26 weeks (A1C treatment difference −0.54%; P < 0.001) [10], the addition of colesevelam HCL to sulfonylurea-based therapy over 26 weeks (A1C treatment difference −0.54%; P < 0.001) [11], and the addition of colesevelam HCL to insulin-based therapy over 16 weeks (A1C treatment difference −0.50%; P < 0.001) [12].

While clinical trials have demonstrated the benefits of colesevelam HCL for T2DM, less information exists regarding real-world, population-based experience. Evidence of real-world effectiveness is important because evidence of efficacy reflected by randomized controlled trials may not be generalizable to broader populations that actually receive treatment in routine clinical practice [13]. Trial eligibility criteria may exclude or under-represent important subgroups of patients such as patients with complex comorbid conditions or certain age groups. Further, medication adherence in trials is often better than in the general population, and in fact in colesevelam HCL trials medication adherence was 93% [10-12], as opposed to the estimated 50% that exists in the broader population [14]. These factors might impact estimates of treatment effectiveness. Therefore, broader evidence is needed to support the benefits of treatment in practice.

The purpose of this study was to examine changes in A1C for patients with T2DM receiving colesevelam HCL using data from a large electronic health record, which reflects the real-world experience with colesevelam HCL for lowering A1C in patients with T2DM. Results from this analysis also demonstrate the extent of concordance between evidence of clinical efficacy from randomized trials and real-world effectiveness. This real-world effectiveness information is particularly useful given the diversity of patient characteristics, risk factors, and other treatments used in everyday practice which are not represented by clinical trial data.

## Methods

### Data and sample

The data and sample were derived from the General Electric (GE) Centricity Clinical Data Services files from 2000–2011. These files are generated by ambulatory health care offices that use the GE Centricity electronic medical record (EMR) and contribute data to the research files. These data represent more than 40 US states and a variety of ambulatory medical practices, including solo practitioners, community clinics, academic medical centers, and large integrated delivery networks [15]. Compared with US Census estimates, higher proportions of patients represented in the data reside in northeastern and mid-western states, while lower proportions reside in southern and western states [16]. The GE electronic health record (EHR) includes information on prescribed medications, lists of medications that the patient reports, patient sociodemographics (age, gender, race, insurance coverage), diagnoses, procedures, and results from ordered lab tests. These data represent information documented in the electronic medical record for the purposes of delivering care. The data were stripped of all 18 unique patient identifiers and did not use any protected health information that could be linked to human subjects. This study was exempt from ethics approval from an institutional review board and informed consent because, according to the US Department of Health and Human Services Exemption 4 (CFR 46.101(b)(4)), the research involved the study of existing data, and the subjects could not be identified directly or through identifiers linked to the subjects.

The sample was a retrospective cohort of patients with T2DM. Patients were included if they had at least one diagnosis of T2DM in the database (250.x0 or 250.x2). Initiation of colesevelam HCL was identified as the first observed prescription or medication list entry in the dataset, and the date of this prescription defined the index date. Patients had to have database activity 395 days before the index date to ensure new colesevelam HCL use. The sample was further restricted to patients 18 years or older on the index date who had a baseline A1C > 7%, as measured by their last A1C value within 90 days prior to starting colesevelam HCL. Patients who had other bile acid sequestrants before the baseline A1C measurement were excluded to isolate the effect of colesevelam HCL treatment on A1C changes. Patients also had to have at least one follow-up A1C measurement between 42 to 210 days after initiation. A period of 395 days prior to the index date was used to define population characteristics and identify other medication use. The follow-up period started at index date and continued for 395 days. The 395 day period for pre- and post-index observation was selected as the maximum period of time that would need to be observed in order to measure chronic medication use, since prescription refills cannot extend beyond 1 year and this 395 day period allows for 1 year plus a 30 day grace period for prescription renewals [16].

### Outcomes

The primary outcome was change in A1C between initiation of colesevelam HCL (i.e., baseline) and three alternative time periods, including 16-weeks (days 42–140), 26-weeks (days 42–210), and 52-weeks (days 42–395) following initiation. The 16- and 26-week endpoints were designed to represent comparative endpoints for clinical trials [10-12]. The 52-week endpoint captured extended glycemic control from colesevelam HCL treatment beyond that observed in most clinical trials. The last observed A1C lab measurement during each interval was used to define the A1C measurement for the corresponding time period.

### Covariates

Population and treatment characteristics were assessed as age, sex, race (i.e., white, black, Asian, other, unknown), insurance type (i.e., commercial, Medicare, Medicaid, self-pay, other, unknown), and region (i.e., midwest, northeast, south, and west). Other treatment use during the baseline and follow-up period were characterized by major drug groups for T2DM, including drug class-level indicators for metformin, insulin, sulfonylureas, thiazolidinediones, DPP-4 inhibitors, and other miscellaneous diabetes drugs.

### Analysis

Descriptive statistics on the final analytic cohort were examined via means and proportions. Change in A1C from baseline to 16-weeks, baseline to 26-weeks, and baseline to 52-weeks was examined via two tailed t-tests. An a priori alpha of 0.05 was selected for statistical significance. The primary analysis considered changes in A1C from baseline for all eligible patients that started colesevelam HCL following an intention to treat (ITT) approach. Unlike claims-based analyses which allow observation of each prescription fill billed to an insurance provider, GE data assume continued treatment by a patient in the absence of a change in prescribing by a provider as documented in the electronic health record, making it more difficult to fully capture medication changes and discontinuation. Given this potential limitation, we felt the ITT analysis appropriate since it did not restrict patients to continued use of colesevelam HCL through the entire measurement period but only required evidence of a prescription at index date.

We also conducted two sensitivity analyses. First, a sensitivity analysis addressed the potential bias of the ITT approach by considering only patients who stayed on colesevelam HCL during the follow-up period, with no evidence of discontinuation up to 30 days before their last relevant follow-up A1C measurement. These patients might be considered analogous to patients in a trial analyzed as “on treatment”, “complete cases”, or “per protocol” (referred to as “on treatment” from here forward). Second, a set of sensitivity analyses considered the effects of other treatments when measuring the colesevelam HCL effect as there are many other T2DM medications on the market and our study design allowed patients to take multiple such medications at any given time. These analyses divide patients into three strata: 1) “Monotherapy” initiators, defined as no other T2DM medication during either baseline or follow-up; 2) “Augment/Switch”, defined as use of at least one other T2DM medication during baseline, and colesevelam HCL was added to the existing treatment or the patient switched treatment to colesevelam HCL; and 3) “New Combination”, defined as no other T2DM medication during baseline but receipt of colesevelam HCL and at least one other diabetes medication during follow-up. Initially, the augment and switch groups were defined as separate groups, but because only 3%-5% (depending on the stratified analysis) of those who were on other T2DM medication switched to colesevelam, the two groups were combined. In defining these groups, a drug was considered to be used during baseline if it was used at least once prior to the baseline A1C measurement. Similarly, a drug was considered to be used during the outcome assessment interval (i.e., 16-weeks, 26-weeks, or 52-weeks) if there was no evidence of discontinuation from the time the drug was started through 30 days before the last A1C measurement during that interval. There are 6 stratified analyses because these analyses were done for both the ITT and the on-treatment approach, and for all 3 time points (16-weeks, 26-weeks, and 52-weeks).

## Results

### Characteristics of the study sample

**Figure 1:**
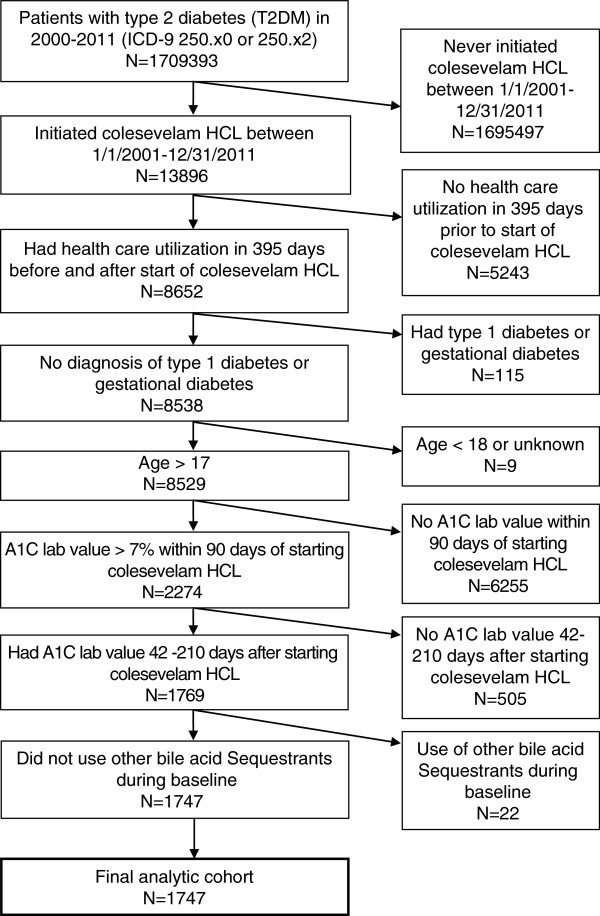
Sample selection.

Of 1,709,393 patients in the GE database with T2DM, 1,747 met inclusion criteria and began treatment with colesevelam HCL (Figure 1). The cohort was 58% female, 38% age 65 and older, and the majority (54%) were white (race information is missing for 34% of patients). This compares with colesevelam HCL trial populations that were, on average, 47% female, 59% white, and with a mean age of 56 years [10-12]. Commercial insurance (26%) and Medicare (36%) were the most common forms of coverage, but the payment type for 35% of patients were unknown. At baseline the mean A1C was 8.25% among all patients prescribed colesevelam HCL. Prior to beginning colesevelam HCL, 29% of patients had previously received insulin, 15% had received metformin (the only biguanide available), and 13% had received sulfonylurea drugs (Table 1).

**Table 1:** Patient characteristics for colesevelam HCL users

**Characteristic**	**All T2DM patients (N = 1747)**
**Age (n, %)**	
18-44 years	132 (7.56%)
45-49 years	149 (8.53%)
50-54 years	208 (11.91%)
55-59 years	299 (17.12%)
60-64 years	300 (17.17%)
65-69 years	263 (15.05%)
70-74 years	195 (11.16%)
75-79 years	126 (7.21%)
≥ 80 years	75 (4.29%)
**Sex (n, %)**	
Female	1018 (58.27%)
Male	729 (41.73%)
**Race (n, %)**	
White	938 (53.69%)
Black	160 (9.16%)
Asian	6 (0.34%)
Other	47 (2.69%)
Unknown	596 (34.12%)
**Insurance Type (n, %)**	
Commercial	454 (25.99%)
Medicare	637 (36.46%)
Medicaid	24 (1.37%)
Self-Pay	10 (0.57%)
Other	8 (0.46%)
Unknown	614 (35.15%)
**Region (n, %)**	
Midwest	350 (20.03%)
Northeast	433 (24.79%)
South	847 (48.48%)
West	117 (6.70%)
**Baseline A1c Values (mean, SD)**	8.25 (1.32)
**Prior T2DM Treatments (n, %)**	
Biguanide	266 (15.23%)
Insulin	506 (28.96%)
Sulfonylurea	231 (13.22%)
Thiazolidinediones	118 (6.75%)
DPP-4 inhibitor	63 (3.61%)
Other Miscellaneous	64 (3.66%)
**Number of Prior T2DM Treatments (n, %)**	
0	808 (46.25%)
1	687 (39.32%)
2	204 (11.68%)
3	42 (2.40%)
≥4	6 (0.34%)

### Change in A1C after initiation of colesevelam HCL

#### Intention-to-treat results

Colesevelam HCL initiation corresponded with an A1C reduction at 16-, 26-, and 52-weeks for the unadjusted ITT analysis (Table 2). For the 16-week endpoint (N = 1385), A1C dropped from a mean of 8.22% to 7.75% (mean change −0.47%; 95% CI -0.53, -0.41). For the 26- and 52-week endpoints (N = 1747), A1C dropped from a mean of 8.25% to 7.81% (mean change −0.44%; 95% CI -0.50, -0.38) and 7.79% (mean change −0.46%; 95% CI −0.52, -0.40), respectively. All the changes were statistically significant (P < 0.0001).

**Table 2:** Mean change in A1C (ITT analyses)

**Time point**	**LOCF within day window**	**Target day**	**Actual day**	**N**	**A1C (%)**		
			**Mean**		**Baseline mean**	**Post mean**	**Change mean (95% CI)**
**Week 16**	42 - 140	112	97.0	1385	8.22	7.75	−0.47 (−0.53, -0.41)
**Week 26**	42 - 210	182	146.2	1747	8.25	7.81	−0.44 (−0.50, -0.38)
**Week 52**	42 - 395	365	297.6	1747	8.25	7.79	−0.46 (−0.52, -0.40)

P < 0.0001 for all based on t-test of change from baseline to last measure within measurement interval; CI: confidence interval.

#### Sensitivity analysis: on-treatment results

Sensitivity analyses considered the potential impact of the ITT assumption when attributing changes in A1C to colesevelam HCL. When restricting the analysis to patients that remained on colesevelam HCL until up to 30 days before their last A1C measurement, we observed reductions in A1C similar in direction to the ITT analysis, but of slightly larger magnitude (Table 3). For the 16-week endpoint (N = 838), A1C dropped from a mean of 8.22% to 7.69% (mean change −0.53%; 95% CI −0.62, -0.44). For the 26-week endpoint (N = 962), A1C dropped from a mean of 8.25% to 7.71% (mean change −0.54%; 95% CI −0.63, -0.45). For the 52-week endpoint (N = 849), A1C dropped from a mean of 8.26% to 7.66% (mean change −0.60%; 95% CI −0.69, -0.51). All of the changes were statistically significant (P < 0.0001).

**Table 3:** Mean change in A1C (On treatment analyses)

**Time point**	**LOCF within day window**	**Target day**	**Actual day**	**N**	**A1C (%)**		
			**Mean**		**Baseline mean**	**Post mean**	**Change mean (95% CI)**
**Week 16**	42 - 140	112	96.1	838	8.22	7.69	−0.53 (−0.62, -0.44)
**Week 26**	42 - 210	182	142.3	962	8.25	7.71	−0.54 (−0.63, -0.45)
**Week 52**	42 - 395	365	287.7	849	8.26	7.66	−0.60 (−0.69, -0.51)

P < 0.0001 for all based on t-test of change from baseline to last measure within measurement interval; CI: confidence interval.

#### Sensitivity analysis that stratify by concomitant T2DM treatments

Another set of sensitivity analyses considered the impact of previous or concomitant diabetes treatments when trying to quantify the colesevelam HCL effect. Since we used real-world data and our design did not restrict patients to the types of other drugs allowed that could also impact A1C measurement, this stratified analysis attempted to minimize this potential bias. To do this, we created the “Monotherapy”, “Augment/Switch”, and “New Combination” treatment cohorts and re-ran the ITT and on-treatment analysis among each of these population subgroups. Results are illustrated in Tables 4 and 5.

**Table 4:** Mean ITT changes in A1C (stratified analyses)

**Stratum**	**LOCF within day window**	**Target day**	**Actual day**	**N**	**A1C (%)**		
			**Mean**		**Baseline mean**	**Post mean**	**Change mean (95% CI)**
Week 16	42 – 140	112					
**Monotherapy**			97.2	387	7.93	7.52	−0.41 (−0.50, -0.32)
**Augment/Switch**			97.2	745	8.26	7.90	−0.36 (−0.44, -0.28)
**New Combination**			96.1	253	8.57	7.69	−0.88 (−1.07, -0.69)
Week 26	42 – 210	182					
**Monotherapy**			145.0	464	7.95	7.61	−0.34 (−0.43, -0.25)
**Augment/Switch**			146.9	939	8.29	7.97	−0.31 (−0.39, -0.23)
**New Combination**			146.0	344	8.55	7.65	−0.90 (−1.08, -0.72)
Week 52	42 – 395	365					
**Monotherapy**			285.9	367	7.89	7.52	−0.37 (−0.48, -0.26)
**Augment/Switch**			300.6	939	8.29	7.96	−0.33 (−0.41, -0.25)
**New Combination**			300.9	441	8.47	7.65	−0.82 (−0.97, -0.67)

P < 0.001 for all based on t-test of change from baseline to last measure within measurement interval; CI: confidence interval.

**Table 5:** Mean on-treatment changes in A1C (stratified analyses)

**Stratum**	**LOCF within day window**	**Target day**	**Actual day**	**N**	**A1C (%)**		
			**Mean**		**Baseline mean**	**Post mean**	**Change mean (95% CI)**
Week 16	42 – 140	112					
**Monotherapy**			96.5	239	7.89	7.51	−0.38 (−0.49, -0.27)
**Augment/Switch**			96.0	448	8.24	7.80	−0.44 (−0.55, -0.33)
**New Combination**			96.1	151	8.66	7.63	−1.03 (−1.31, -0.75)
Week 26	42 – 210	182					
**Monotherapy**			141.5	268	7.90	7.55	−0.35 (−0.46, -0.24)
**Augment/Switch**			143.7	511	8.28	7.87	−0.40 (−0.51, -0.29)
**New Combination**			139.3	183	8.69	7.51	−1.18 (−1.46, -0.90)
Week 52	42 – 395	365					
**Monotherapy**			283.7	188	7.83	7.40	−0.43 (−0.59, -0.27)
**Augment/Switch**			289.2	439	8.29	7.84	−0.45 (−0.57, -0.33)
**New Combination**			288.3	222	8.55	7.52	−1.03 (−1.26, -0.80)

P < 0.0001 for all based on t-test of change from baseline to last measure within measurement interval; CI: confidence interval.

The A1C changes in each group remained statistically significant (P < 0.001). The results follow a similar pattern to previous analyses, although the A1C reductions were larger for the “New Combination” group as opposed to the “Monotherapy” and “Augment/Switch” groups. For example, across the 16-, 26-, and 52-week endpoints in the ITT analysis (Table 4), the mean reduction in A1C was between −0.34% to −0.41% for the newly treated colesevelam HCL monotherapy group, -0.31% to −0.36% for the colesevelam HCL augment or switch group, and −0.82% to −0.90% for the newly treated group that included colesevelam HCL combined with another medication. Similarly, in the on-treatment analysis (Table 5), the mean reduction in A1C was between −0.35% to −0.43% for the newly treated colesevelam HCL monotherapy group, -0.40% to −0.45% for the colesevelam HCL augment or switch group, and −1.03% to −1.18% for the newly treated group that included colesevelam HCL combined with another medication. Comparing the ITT results with the on-treatment results, only modest differences in the magnitude of the A1C change were observed. Further, the mean change in A1C was relatively consistent over time for both the ITT and the on-treatment analyses.

## Discussion

In this analysis of real-world use of colesevelam HCL, we observed a mean reduction in A1C of 0.47% at 16-weeks, 0.44% at 26-weeks, and 0.46% at 52-weeks using a conservative ITT approach. These analyses support the real-world benefits of colesevelam HCL for managing diabetes and can be compared with clinical trials, where A1C improved by 0.3% to 0.4% over 16- to 26-weeks with colesevelam HCL, as compared with an increase of approximately 0.1% to 0.2% for placebo (placebo-adjusted treatment difference of 0.50% to 0.54%) [10-12].

Our primary analysis used an ITT approach. In the context of clinical trials, ITT analyses are considered a preferable approach because randomization is preserved and results are believed to be more reflective of real-world practice since not all patients given a treatment will tolerate it or continue treatment [17]. The ITT analysis ignores non-adherence, discontinuations, and other changes in treatment such as switching between diabetes medications. The last observation carried forward approach used with ITT handles missing data by carrying forward the last observed measurement as the planned endpoint. The ITT approach generally is not favored in the context of retrospective observational studies because there is no need to preserve randomization and data generally are rich enough to measure time-dependent drug exposure. But, the ITT principle is important in the context of our real-world data because our data were prone to problems with limited ability to measure drug exposure, and outcome measurement (i.e., A1C) was inconsistent with regard to timing and consistency. The ITT approach therefore allowed us to conservatively estimate the treatment effect without being overly restrictive in our population selection on the basis of medication use patterns and outcome measurement. Still, these analyses are subject to biases that might be introduced by making assumptions about ongoing medication exposure or carrying forward A1C measures that may be too far removed from treatment. These biases may be more pronounced in the 26 and 52 week outcomes which are further removed from baseline observation.

To overcome some of the concerns related to use of an ITT approach, our sensitivity analyses considered whether patients continued to take their colesevelam HCL and the potential effect that other medications might have on the outcome of interest (i.e., A1C change). For example, in our “on treatment” sensitivity analysis, we restricted the analysis to patients that remained on treatment until at least 30 days before their last A1C measurement. This assumes ongoing medication use and we would expect the A1C improvements to be larger in this analysis as compared with the more conservative ITT analysis. For the on treatment analysis, we observed a mean reduction in A1C of 0.53% at 16-weeks, 0.54% at 26-weeks, and 0.60% at 52-weeks. These A1C improvements were of greater magnitude than the reductions observed in the ITT analysis, as expected. While this does not fully address the issue of adherence since the electronic medical record data is limited in terms of the insight it can provide regarding medication adherence, the fact that the medication was still active for these patients and we in turn observed greater A1C improvements when restricting to active medication profiles is an important finding.

Relevant RCTs have all added colesevelam HCL to an existing treatment (e.g., insulin, metformin, or sulfonylurea) and found greater improvements in A1C with the addition of colesevelam HCL compared with groups that added placebo to their existing treatment [10-12]. In the real-world, prior studies have shown that on average patients with T2DM use 1.6 prescription medications to manage their diabetes [18]. In our sensitivity analyses that considered the potential impact of other treatments, we found the A1C reductions to be largest for the group that newly initiated colesevelam HCL with another diabetes medication. For this group, the A1C reductions were −0.82% to −0.90% in the ITT analysis and −1.03% to −1.18% for the on-treatment analysis. These A1C reductions likely reflect both the colesevelam HCL effect and the beneficial effects of other medication(s), making it difficult to isolate the effect of colesevelam on A1C reductions. But, for context, this compares with a clinical trial conducted in patients with early T2DM who had not previously received treatment [19]. In this 16-week trial, initial combination therapy with metformin and colesevelam HCL reduced mean A1C levels by 1.2% compared with a 0.8% reduction in mean A1C for the metformin plus placebo group (P = 0.0035). The mean difference in A1C reduction between these groups (−0.4%) is a reflection of the additional benefit of adding colesevelam HCL. Interestingly, in our subgroup of patients who newly initiated colesevelam HCL with no other diabetes medication, we observed an A1C reduction of 0.41% at 16-weeks in our ITT analysis. Differences observed in our real-world analysis parallel the differences reflected by trial data, providing additional evidence regarding the incremental improvements in A1C that may be realized with colesevelam HCL.

Several limitations are inherent in the use of observational data for research and in our analysis. While the ability to capture laboratory measurements is an important strength of these data, we were unable to account for measurements that might have been made outside of the clinics contributing data to the GE record. Additionally, unlike clinical trials which allow for regular scheduled laboratory testing, we had no control over the timing of laboratory measurements relative to the start of colesevelam HCL. For measurement of exposure to colesevelam HCL and other treatments, we relied on the medication table which includes both patient-reported medications as well as prescriptions written. Using the prescription written table may have been a more accurate approach, but then we could have missed use of colesevelam HCL from other providers not contributing data to the GE health record. Further, the electronic medical record does not reflect medication dispensing (as claims data does) or any aspect of patient adherence, so we had to assume that if a medication was active and there was not stop date then the patient was likely still on the medication. This assumption could overestimate medication adherence, and likely this overestimation would be most reflected by our “on treatment” sensitivity analysis. Given the high degree of non-adherence to diabetes treatments observed in prior studies, our results are likely conservative and A1C effects could be more pronounced if adherence could be better accounted for in our analysis. While our data reflect an electronic medical record system representing broad US coverage and many different types of populations and insurance sources, the clinics using this record system might be different from other healthcare settings and this could influence the types of patients represented in this study. Prior studies suggest that the GE dataset may have better representation of younger patients and females [20], and we observed a higher representation of females in our sample (58%) as compared with colesevelam HCL trials (47%) [10-12]. Finally, since our study focused on colesevelam HCL users rather than making a comparison with non-users or with other treatment groups, we did not adjust for covariates besides other medication use in our analysis. Other factors may directly or indirectly influence A1C (e.g., diet, exercise, weight change, comorbid conditions, etc.), but since we used a pre-post design and these factors were either not measureable or believed to be fixed, they were not adjusted in our analysis. Exploratory analysis of covariance (ANCOVA) revealed mixed effects of the impact of age and sex across each of the time periods studied. Additional analyses with larger sample size might explore how colesevelam HCL-related A1C changes differ among subgroups.

## Conclusions

In this study of real-world patients with T2DM treated with colesevelam HCL, A1C improved by 0.44% to 0.47%. This reduction in A1C is similar to the A1C improvements reported in RCTs, supporting the real-world effectiveness of colesevelam HCL in managing T2DM.

## Competing interests

The study was funded by Daiichi-Sankyo, Inc (DSI), the makers of colesevelam HCL (Welchol). Article processing charges were paid by DSI. Drs. Ye and Qian are employees of DSI, and Drs. Hansen, Farley, Maciejewski, and Powers received research funding from DSI. During the past 5 years, Drs. Hansen, Farley, and Maciejewski also have received research support from Novartis and Takeda Pharmaceuticals. Dr. Maciejewski has received consulting funds from the Surgical Review Corporation and the University of Minnesota, and owns stock in Amgen.

## Authors’ contributions

All authors contributed to the design of the study and protocol development. CQ conducted the data analysis. RH, JF, MM, and XY critically reviewed the statistical analyses. RH drafted the manuscript. All authors read and approved the final manuscript.

## Pre-publication history

The pre-publication history for this paper can be accessed here:

http://www.biomedcentral.com/1472-6823/13/24/prepub
